# Comparative Analysis of the Efficiency of Medicinal Plants for the Treatment and Prevention of COVID-19

**DOI:** 10.1155/2022/5943649

**Published:** 2022-12-10

**Authors:** Viktor Kamkin, Aidana Kamarova, Baurzhan Shalabayev, Assyltas Kussainov, Marat Anuarbekov, Serik Abeuov

**Affiliations:** ^1^Department of Agrotechnology, NJSC Toraighyrov University, Pavlodar 140008, Lomov 64 St, Kazakhstan; ^2^Department of Biology and Ecology, NJSC Toraighyrov University, Pavlodar 140008, Lomov 64 St, Kazakhstan; ^3^LLP AlemAgro Holding, Pavlodar 140000, Lugovaya 16 St, Kazakhstan

## Abstract

The COVID-19 pandemic has once again prompted people to resort to the remedies of folk and alternative medicine. Medicinal plants, because of their chemical composition, pharmacological properties, and the action of biologically active substances, can stop and relieve the symptoms of the disease. The purpose of the work is a comparative flora analysis of medicinal plants to identify the most prospective plant and further production of a remedy for the avoidance, treatment, and rehabilitation of COVID-19. The search for prospective medicinal plants was performed by analyzing the literature in online databases: Web of Science, Scopus, Google Scholar, and PubMed, including official WHO media sites. According to recent studies related to COVID-19, a significant number of medicinal plants with anti-inflammatory, antiviral, and immunostimulatory effects have been identified. A comparative study of nine medicinal plants was conducted to determine the most suitable medicinal plant to treat coronavirus infection. According to the results of the comparative analysis, *Chamaenerion angustifolium* Seg. showed itself as the most prospective medicinal plant with the greatest pharmacological effect compared with other types of medicinal plants. Its therapeutic properties allow physiological relief of 18 symptoms of coronavirus infection. It is advisable to conduct further clinical trials for the treatment and rehabilitation of COVID-19 using preparations from this plant.

## 1. Introduction

The current coronavirus pandemic has shown the importance of providing the nation with effective and safe domestically produced medicines. In this regard, herbal medicines with a wide range of therapeutic action and several advantages in comparison with medicines of synthetic origin are of particular relevance. Herbal medicines are characterized by a relatively low risk of allergy development, a milder therapeutic effect, and safety. The main selection criteria for herbal raw materials as a source of pharmacologically active compounds are high amount of active agents, availability of raw materials in nature, and simple technology of their cultivation.

The rapid spread of coronavirus infection has boosted the market demand for medicinal plant raw materials. There was a problem of shortage of medicines. The shortage of drugs was also related to the pandemic because the prices on the world market were inflated, and there were difficulties with transportation due to closed borders. The anxiety caused by the side effects of chemical drugs, the desire to create a healthcare system with a more personalized approach, and the public's wide access to health information all contribute to the wider use of alternative medicine. In addition, there is a significant list of residual effects after COVID-19 symptoms have occurred, and in some patients, they do not go away after several months. There are no drugs that can alleviate these symptoms yet. Therefore, the population has resorted to traditional and alternative medicine.

Plants have been used in folk medicine since ancient times. As is well known, plants contain chemical substances, the action of which is directed to various processes, entering complex interactions with the organism. Medicinal properties of plants are caused by the presence in their organs of active substances (alkaloids, flavonoids, glycosides, vitamins, tannins, and coumarin compounds) that have a physiological effect on human and animal organisms or have biological activity against pathogens of various diseases [1, 2]. Plants produce aromatic substances, most of which are phenols, and their oxygen-substituting derivatives are useful for health support. For example, one medicinal plant can replace several synthetic drugs and be used in the treatment of diseases of various organs and systems, both the main and related diseases [3]. It has long been established that plants can have different effects on the human body.

Alkaloids are plant defense mechanisms against microorganisms, insects, and animals. Some biologically active plant compounds are active against strains of viruses, even those that have adapted to antibiotics.

Flavonoids have been reported to be able to prevent the induction of COX by prostaglandins in inflamed tissues. Apigenin is known to reduce the flow of lipids necessary for pain signal transmission. Thus, flavonoids reduce inflammatory pain by inhibiting receptors and signaling pathways [4].

The work by da Silva et al. [5] presents the results of the molecular docking of 171 essential oil components with the main protease SARS-CoV-2. The scientists hypothesized that essential oil components might interact with key protein targets of the disease. As a result, they found that essential oils can potentiate the effects of other antiviral agents or they can provide some relief from the symptoms of infection.

Currently, there is still an active search for effective drugs to treat COVID-19 and eliminate residual symptoms of the disease. Treatment guidelines are constantly changing, the virus mutates, and new symptoms of the disease appear. The effect of the disease depends on the immune state of the body; complications arise in the presence of concomitant chronic diseases [6]. Herbal remedies have low toxicity compared with synthetic drugs and are used in the pharmaceutical industry because of their powerful biological activity. Because of the action of complex reactions in the body, they contribute to a quick recovery and an easy course of the disease.

The purpose of the work is a comparative flora analysis of medicinal plants to identify the most prospective plant and further production of a remedy for the avoidance, treatment, and rehabilitation of COVID-19.

## 2. Materials and Methodology

**Figure 1:**
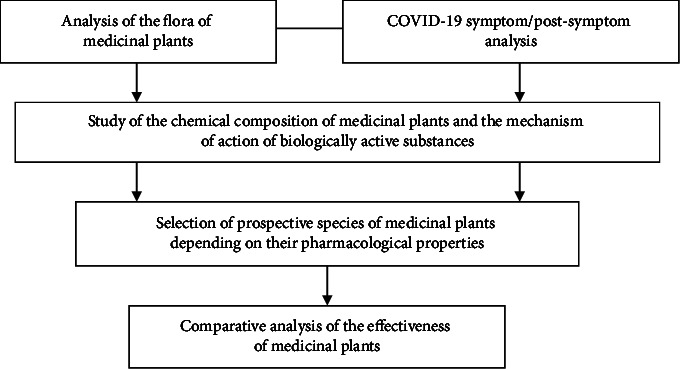
Research scheme.

The methodology of this work is based on the recent literature review on the action of biologically active substances in plants, their chemical composition and pharmacognostic properties, and their effect on coronavirus infection (Figure 1).

Data were searched by analyzing the literature in online databases: Web of Science, Scopus, Google Scholar, and PubMed, including official WHO media sites. The experience of traditional medicine in treating coronavirus infection with plant-based preparations was also critically analyzed, looking for scientific confirmation in scientific publications of the effectiveness of the proposed medicinal plants and their preparations.

## 3. Result and Discussion

Coronaviruses (*Coronaviridae*) belong to the family of RNA-containing viruses capable of infecting humans and some animals and are included in pathogenicity group 2 along with other members of this virus family [7].

The main route of transmission is direct or indirect exposure to the respiratory tract. The cause of virus can infect other people, including during the incubation stage of the infection. The virus is transferred by airborne, open, and contact routes. Factors of transmission are air, that is to say, being in closed and poorly ventilated rooms, food, as well as contaminated objects and surfaces. The incubation period averages from 2 to 14 days [8].

The infectious process goes on in several stages. The initial stage requires viral reproduction, led by mild symptoms and activation of the inherent resistant response. Adaptive protection is turned on, and either the infection is passed, the virus clears up, or it becomes acute or chronic. The clinical range can differ depending on the viral load and the immune position of the patient [9]. Patients with chronic diseases, hypertensive patients, diabetics, and the elderly with comorbidities are at risk for developing acute coronavirus [10, 11].

COVID-19 disease affects various parts of the human body, producing serious illnesses.  Table 1 details the effects of the virus on the internal organs: the heart, lungs, vessels, kidneys, brain, central nervous system, and skin of people.

We would especially like to focus on the Delta strain (Delta COVID-19 (B.1.617.2 or AY.1)). It was early described in India in October 2020, and afterward, the strain was again found in other countries [31]. The WHO considers the Delta strain to be the most dangerous compared with the common coronavirus [32]. The mutated strain of coronavirus causes slightly different symptoms in infected people than the original coronavirus. Symptoms typical of SARS-CoV-2 such as loss of smell and taste are not observed. The following symptoms are distinguished: headache, rhinitis, sore throat, fever, and cough [33]. Transmission occurs in different age groups, has a high rate of spread, the incubation period is much shorter, ranging from 1 to 3 days, and an increased viral load is noted [30].

The Delta strain raises concerns among doctors and scientists because it binds more easily to lung cell receptors and also resists monoclonal antibodies designed to treat the disease [34]. An important feature of the Delta strain is that it can cause mucormycosis and aspergillosis, invasive fungal infections caused by fungi *Aspergillus fumigatus,* and *Rhizopus arrhizus* [35]. Fungal infections affect the central nervous system, eyes, joints, and other organs and develop as a result of antibiotics, corticosteroids, and prolonged artificial ventilation [36].

Since ancient times, medicinal herbs have been used to incur different illnesses [37]. It required special plants to provide infections and viruses such as colds and flu, to strengthen the human immune system because the active substances of these plants have a significant therapeutic effect. Moreover, chemicals isolated from plants, such as aspirin (from willow bark) and morphine (from opium), are used to produce various medicines. Diseases such as diabetes, hypertension, arthritis, and oncology are treated with medicinal plants [38–40].

Moreover, medicinal plants have an antiviral effect and increase the body's resistance to epidemic diseases, which is very relevant at present [41]. The action of medicinal plants has already shown promising results in studies as an effective treatment to treat severe acute respiratory syndrome (SARS) and Middle East respiratory syndrome (MERS) [42]. In addition, the combination of traditional and folk medicine methods has reduced the adverse effects of glucocorticoids, antibiotics, and antiviral drugs in the cure of SARS [43]. Medicinal plants can be an effective weapon for treating coronavirus; however, appropriate clinical trials are recommended.

Analysis of the flora of medicinal plants showed the following plants to treat coronavirus infection (Table 2).

### 3.1. *Nigella Sativa* L. (Black Cumin)

*Nigella Sativa* L. has long been used in folk medicine. The use of black cumin seeds is especially valuable; they have several therapeutic properties and are used to treat hypertension, asthma, liver diseases, stomach disorders, oncology, immune disorders, neurological, and many other diseases [71–73]. In studies on animals, the positive effect of extracts and oil of *N. Sativa* in different diseases, including arthritis, hypertension, bacterial and viral infections, type 2 diabetes, asthma, neurological, and dermatological diseases [74–76]. Black cumin is being studied for comparison with drugs such as hydroxychloroquine, chloroquine, azithromycin, arbidol, remdesivir, ribavirin, chloroquine phosphate, lopinavir/ritonavir, and favipiravir since its use in antiviral therapy has been proven in studies [77–79]. According to recent data, black cumin can be used as a new drug against COVID-19. This is mentioned in research carried out by Shad et al. [79]; compounds obtained from *N. Sativa*, in specific thymoquinone, can inhibit the capacity of SARS-CoV-2 to attach to introduce cell receptors and reproduce inside the cell [80].

Studies conducted by Koshak et al. showed that taking *N. sativa* supplements showed faster recovery from the disease compared with classical treatment [81].

### 3.2. *Glycyrrhiza glabra*, *Glycyrrhiza uralensis* (Licorice)

In medicine, licorice is used as an expectorant, cough suppressant, laxative, cardiovascular, respiratory, immunological, anti-inflammatory, analgesic, and antipyretic [48]. *Glycyrrhiza glabra* contains more than 20 triterpenoids and about 300 flavonoids, among them glycyrrhizin, a saponin compound isolated from the roots [82]. Several types of research have demonstrated that glycyrrhizin has shown a significant inhibitory effect on the influenza virus and is an effective antiviral compound against the hepatitis C virus (HCV) [83]. A clinical trial conducted in 2003 showed the potential antiviral activity of glycyrrhizin against 2 clinical isolates of coronavirus infection (FFM-1 and FFM-2) from patients [84]. An *in vitro* research by Shahrajabian et al. demonstrated an antiviral effect; glycyrrhizin suppressed the virus associated with SARS and has been proposed to treat SARS [85].

### 3.3. *Artemisia annua* L

The main bioactive compound of Artemisia is Artemisinin a sesquiterpene lactone having a special peroxide bridge which is separated from wormwood and is used as a remedy against malaria [86]. Artemisinin and its synthetic derivatives have demonstrated significant effects against other parasitic diseases [87], several different types of cancer [88] *in vitro*, and individual scientific experiments. *A. annua* extract demonstrated antiviral activity *in vitro* against coronavirus in 2005 [89]. Artemisinin-rich* A. annua* extracts are being considered in current stage 2 clinical trials against COVID-19 [90]. Fuzimoto et al. reported that *A. annua* can remodulate the host's innate and adaptive immunity and help reduce cytokine storms and symptoms of coronavirus infection [91].

### 3.4. *Artemisia vulgaris*

Artemisia contains secondary metabolites such as flavonoids, terpenoids, saponins, and polysaccharides [92]. Arglabin, a sesquiterpene lactone located mainly in all wormwood species, shows a marked anticancer effect against different tumor cell lines [53]. A lot of analogs, such as artesunic acid, artelic acid, and artemether, can be produced and are sufficient against multidrug-resistant malaria [93]. Artemisia has found several therapeutic uses in folk medicine that are related to the gastrointestinal tract, such as ulcers, indigestion, and liver problems [94]. The plant is also used to treat worm infestation, anxiety, epilepsy, vegetative neurosis, insomnia, neurasthenia, and general irritability. The plant essential oil is expected to have a sort of pharmacological activity, with an increased range of bioactivity, because of the metabolites' action of auxiliary chemical components that lead to their functioning through different modes of action [95]. Leaf powder or paste is applied to provide skin illnesses and has analgesic effects [52].

### 3.5. *Colchicum autumnale*

*Colchicum autumnale* contains colchicine is an alkaloid and is one of the best-known natural products appealing to a class of organic compounds found as tropines. The therapeutic properties of colchicine have been identified for a long time; it is used to treat gout but has attracted increased attention because of its activity as an antimitotic agent [96]. New therapeutic uses of colchicine, in addition to the treatment of gout, are currently being explored. Scientists Schlesinger et al. conducted ten clinical trials of colchicine to deal with SARS-CoV-2 disease [97]. According to the results of the studies, data on its anti-inflammatory and antiviral properties were published, the mechanism of activity of colchicine through the tubulin-colchicine complex was described, and the process of colchicine action in the fight against COVID-19 was explained [54].

### 3.6. *Inula helenium*

Preparations from the rhizomes of *Inula helenium* have expectorant and anti-inflammatory effects, improve appetite, reduce intestinal peristalsis, and reduce the secretion of gastric juice. It is believed that the main biologically active substance of elecampane is alantolactone and related terpenoids. The rhizomes and roots are used to produce the drug allanthone, which is used to treat gastric and duodenal ulcers. According to Gierlikowska et al., the use of elecampane to treat inflammatory respiratory illnesses (e.g., cough, fever, bronchitis, rhinitis, and flu) [56] was confirmed [57].

### 3.7. *Aloe vera*

It is one of the most researched and used medicinal plants all over the world, and its pharmacological properties are known. In traditional medicine, the useful properties of aloe are used as a laxative; it increases the peristalsis of the colon in small doses, improves digestion, and increases appetite [58]. *Aloe vera* ethanol extract (AVE) has been described to have considerable action against the influenza virus [98]. *Aloe vera* consists of several individual compounds contained in the antiviral activity: quercetin, catechin hydrate, kaempferol, acemannan, azidothymidine, acyclovir, aloin, emodin [99]. Emodin has antiviral activity against some types of viruses such as human cytomegalovirus, herpes simplex virus type 1, and poliovirus [100]. *Aloe vera* contains minerals including Ca, Mg, Na, K, Fe, Co, and Zn. There is evidence that Zn^2+^ inhibits coronavirus RNA and polymerase action *in vitro*, and zinc ionophores stop the reproduction of these viruses in cell culture [101].

### 3.8. *Filipendula*

Due to the presence of ascorbic acid, the plant has an immunomodulatory and bracing effect. The flowers of the plant contain anticoagulant heparin, so *Filipendula* is recommended for use in varicose veins and thrombophlebitis. The presence of salicylates explains the anti-inflammatory activity, so an infusion of *Filipendula* flowers is used as an astringent for the treatment of stomach disorders. Preparations from the rhizome of *Filipendula* are used to treat various skin diseases, to wash purulent wounds, ulcers, furuncles, and boils [102], in the treatment of oral diseases, with eczema of extremities, trophic ulcers, hemorrhoids, itchy dermatoses, bedsores, scabs, diaper rash [103]. Infusion of stem tops with leaves and flowers of *Filipendula* is used for gout, rheumatism, stomach pain, as a diuretic, as a diaphoretic, for heart disease, choking, headache, diarrhea, for dysentery, for hysterical convulsions, pain in the stomach, and intestines, in the chest, in the throat [59, 60]. It has been experimentally established that medications of *Filipendula* reduce blood pressure by 40% within 20 minutes [104]. The flowers and herbs of *Filipendula* are used in diseases of the upper respiratory tract, as a diaphoretic, in bronchial asthma, and as an antispasmodic agent. They have a sedative effect, and they are prescribed for hypertension, epilepsy, neurasthenia, hypochondria, and other neuroses, such as sleeping pills [61–65].

### 3.9. *Chamaenerion angustifolium* Seg. (L.) Scop. (Ivan-Tea, Kapor Tea)

In *Chamaenerion angustifolium* Seg., almost all the organs (both generative and vegetative) have medicinal or nutritional properties. Corresponding to R. Valov in the chemical composition of *Chamaenerion angustifolium* Seg. there are slight variations (in the shoot part of the plant, containing leaves, stems, and inflorescence). The above-ground part of the plant is of greater interest for research. The peculiarities of its chemical composition are as follows: proteins (12.2–16.4%), mucus (polysaccharides which are easily hydrolyzed) – 8.8–19.4%, cellulose-13.1–26.0%, tannins-6.1–10.1%, anthocyanins-1.0–1.8%, lignin-8.7–13.8%, chlorophyll-5.1–8.0 mg/l, chlorophyll b-9.3–13.6 mg/l, carotene-3.6–7.6 mg %, rutin-16027.72 mg %, vitamin C-56.4–225.1 mg % [105].

Data on the chemical composition of *Chamaenerion angustifolium* Seg., indicate its high content of antioxidants and other biologically active substances that cause its therapeutic properties. The absence of caffeine in it expands the scope of its application to a wide range of age groups, including patients with hypertension, because the caffeine helps narrow blood vessels and reduce the risk of thrombosis. The above data confirm the high nutritional value of *Chamaenerion angustifolium* Seg. Thus, tannins, the content of which in *Chamaenerion angustifolium* Seg. is up to 20%, are represented by tannin, which has an anti-inflammatory effect. This effect is especially valuable for people with gastrointestinal diseases [67].

Equally important is the property of tannin to bind and remove toxic metals from the body. *Chamaenerion angustifolium* Seg. contains a lot of quercetin as well as kaempferol. These substances strengthen blood vessels, preventing them from becoming brittle. They also protect cells from oxidative stress, thus preventing the body from aging prematurely and getting cancer. Kaškonienė et al. identified about 50 different compounds in the essential oil of *Chamaenerion angustifolium* Seg. [106, 107].

Among vitamins, the above-ground part of *Chamaenerion angustifolium* Seg. is especially rich in vitamin C. Its content is three times higher than in citrus fruits. This vitamin is especially important for preventing aging and cancer processes, as well as improving the absorption of iron and strengthening the walls of blood vessels [108].

Group B vitamins in *Chamaenerion angustifolium* Seg. are represented by thiamine, nicotinic acid and folic acid. These vitamins are known to be anti-stress, normalize digestion, improve brain cell function, and optimize protein and fat metabolism. Leaves and flowers of *Chamaenerion angustifolium* Seg. contain sufficient amounts of iron (23 mg per 100 g of raw materials), which is essential for human blood hemoglobin, the synthesis of immune system cells, and the digestive and nervous systems. The high content of manganese (16 mg) and copper prevents the development of anemia, has a beneficial effect on the spleen and liver, supports bone structure, improves memory, and supports growth processes [109].

In addition, the plant is rich in magnesium, molybdenum (0.44 mg), boron (6 mg), potassium, nickel (1.3 mg), lithium, calcium, titanium (1.3 mg), sodium, and other elements. They participate in metabolic processes, are important for muscle function, heart rate, and bone structure, and perform many other important functions in the human body. Different parts of *Chamaenerion angustifolium* Seg. contain essential oils, including limonene, eugenol, benzaldehyde, phellandrene, 3-hexene-1-ol, camphene, linalool, and others [110].

Depending on the studies that were carried out by the scientists Adamczak et al. [68], traditional uses of *Chamaenerion angustifolium* Seg. in phytotherapy include insomnia, headaches, tremors, anemia, infections, delirium, and colds [111]. *Chamaenerion angustifolium* Seg. is required as an antiphlogistic and antiseptic agent to treat mycoses, ulcers, skin rashes, minor burns, wounds, and inflammation of ENT diseases [36, 69]. The aerial parts of *Chamaenerion angustifolium* Seg. (herb and leaves) are used to treat the disorders of stomach and prostate inflammation, as well as the liver. They also are used for kidney and urinary tract diseases. In general therapeutic practice, *Chamaenerion angustifolium* Seg. has anti-inflammatory, antiproliferative, immunomodulatory, antimicrobial, and antioxidant effects [68]. It is also used to care for wounds and various skin and mucous membrane disorders [112].

As Kadam et al. report, the use of *Chamaenerion angustifolium* Seg. extracts before influenza virus disease decreased mortality and extended mean survival time [113]. These effects were even more striking when the virus appeared seven days after the last administration of the extract. Extracts of the plant also exhibit antitumor properties, including inhibition of human prostate epithelial cells by PZ-HPV-7 [114, 115].

Lasinskas et al. conducted research to evaluate the chemical composition, antioxidant and antibacterial activity of ethanol-water extracts (EVE) of *Chamaenerion angustifolium* Seg. as a valuable source of bioactive substances with antioxidant and antimicrobial characteristic, as well as *in vitro* evaluation of penetration into human skin of certain EVE compounds and their accumulation in the skin [116]. The study made it possible to assess the extent to which the active substances of the plant can be useful in protecting not only the skin surface and its deep layers but also the surrounding tissues from oxidative stress and bacterial infection. According to the study, the pharmacological activity of *Chamaenerion angustifolium* Seg. is due to the content of several biologically active compounds such as phenolic acids, including benzoic acid derivatives such as GA, 3,4-DHB, 4-HB, and cinnamic acid derivatives such as CA [117]. Phenol acids and other antioxidants are considered valuable therapeutic ingredients with antioxidant and antimicrobial properties in topical preparations [13, 118].

When using the herb *Chamaenerion angustifolium* Seg., contraindications and other significant side effects have not been identified. As with any herb, in rare cases, individual intolerance is possible. Also, prolonged use may cause minor gastrointestinal disturbances, passing immediately after stopping it.

The unique biochemical composition determines the variety of medicinal properties of *Chamaenerion angustifolium* Seg.  Table 3 presents a comparison of the main symptoms of COVID-19 and the medicinal properties of *Chamaenerion angustifolium* Seg. contributing to their elimination.

To identify the medicinal plant most suitable to treat coronavirus, a comparative study among nine medicinal plants was performed. The data are demonstrated in Table 4.

According to a comparative analysis, *Chamaenerion angustifolium* Seg. has the greatest pharmacological effect compared with other types of medicinal plants.

The high pharmacological activity of *Chamaenerion angustifolium* Seg. and a wide range of its pharmacological action, along with excellent taste and aromatic properties, are the reason for its demand in the market of herbal medicines. The modern market price of fermented *Chamaenerion angustifolium* Seg. is 19 thousand tenge/kg, which at a yield of 4.3–12.4 cwt/ha allows for an average income of 15865 thousand tenges per hectare. One picker manually collects 20–25 kg of leaves per day, which in terms of dry weight is 4-5 kg. A 1-hectare average yield of 8.35 c/h requires 36 man-days of work to collect plant raw materials. The limitation of the leaves harvesting season to a period of two weeks from the beginning of budding to full flowering provides 3 jobs per hectare per season.

The technological process of production of fermented *Chamaenerion angustifolium* Seg. from leaves of *Chamaenerion angustifolium* Seg. includes collection, drying, twisting, fermentation, drying in an oven, and air drying.

Taking into account the simplicity of the technological process of harvesting and processing, including fermentation, the *Chamaenerion angustifolium* Seg. is a promising medicinal plant for wide implementation into production and cultivation in rural areas. This will create jobs and seasonal income for the local population and provide the pharmaceutical market with effective herbal medicine to alleviate the symptomatic course of COVID-19.

## 4. Conclusions

According to the results of comparative analysis of the flora of medicinal plants among plants with antiviral effects, *Chamaenerion angustifolium* Seg. is the most suitable for the treatment of COVID-19.

From the review of literary sources, it follows that *Chamaenerion angustifolium* Seg. is a medicinal plant raw material with a high potential for biological activity, which should be used in pharmacy as it possesses all medicinal properties for the cure and rehabilitation of patients with coronavirus. Given the wide range of medicinal properties of *Chamaenerion angustifolium* Seg., it is necessary to further research in scientific experiments and implementation in the treatment protocols against the virus.

In conditions of shortage of medicines, it is necessary to introduce in the development of wider use of *Chamaenerion angustifolium* Seg. Additional economic advantages are the prevalence of the plant in the region (eliminates the cost of transportation), unpretentiousness (does not require special care during cultivation), not require special processing (all parts of the plant are used) does not require extraction of pharmacologically active substances in the production of drugs. The possibility of using *Chamaenerion angustifolium* Seg. will create new jobs and give an impetus to the economic development of the region.

A lot of studies are devoted to the potential effect of medicinal plants and herbal preparations on the treatment and prevention of COVID-19 [122–124]. For example, the research of Sankar et al. reported the presence of potent phytocompounds in the composition of some medicinal plants. The study showed that the identified phytocompounds could be considered promising medicinal compounds [125]. Shree et al. attempted to recognize natural phytochemicals from medicinal plants to use against COVID-19 using molecular docking and molecular dynamics studies. Based on their findings, they suggest that active phytochemicals from medicinal plants might inhibit Mpro SARS-CoV-2 [126]. Hu et al., in their research, revealed that the citrus flavonoid rutin could affect the assembly of viral functional proteins and suppress inflammation in the host [127].

The data presented above clarify that it may recommend medicinal plants and their biologically active substances for use as complementary and alternative medicine or may be potential sources for the discovery and development of drugs against COVID-19, as well as other viral infections.

However, complementary and alternative medicine is often undervalued by the medical system. But for many people, complementary herbal remedies and medicines are the main source of medical care, and sometimes the only one, because of their territorial and financial accessibility. In addition, because of the COVID-19 pandemic, the affordability of most herbal remedies makes them more attractive than classic medicines. Undoubtedly, remedies of herbal origin require research, the main difficulty in studying them is and remains a large list of studies. A more extensive study of *Chamaenerion angustifolium* Seg. and other medicinal plants requires the study of their biological and pharmacological properties, as well as technological features of processing, conducting clinical trials to get high-quality, safe drugs, and working out the best ways of its reception considering the characteristics of patients. At this stage of research, there is a need for cooperation between botanical and medical research institutions. The pharmacognostic studies that we have conducted need further clinical validation.

Funding limitations and the inability to transfer results to real clinical settings are significant limitations for researchers, so there is an urgent need to continue research work to explain the potential use of medicinal plants and herbal medicines for the treatment and prevention of COVID-19.

## Figures and Tables

**Table 1:** The main influence of COVID-19 on humans.

No.	Organs of defeat	Consequences COVID-19
1	Heart	Myocarditis, cardiac rhythm disorders, thrombosis, embolism, acute coronary syndromes [12], acute heart failure [13], and BP dysregulation (hypertensive crises, and hypotension) [14, 15]
2	Lungs	Atypical pneumonia, hypoxia, acute respiratory failure syndrome, cytokine storm, pulmonary fibrosis, decreased transparency of lung tissue, and so-called “frosted glass” symptoms [16–18]
3	Vessels	General vascular endothelial inflammation, thrombosis, cardiac abnormalities, pulmonary embolism, DIC syndrome blood thickening, acute coronary syndrome, myocardial infarction, and stroke [10, 19–21]
4	Central nervous system	Disturbance of sense of taste and smell, panic attacks, insomnia, anxiety, depression [22, 23], confusion, decreased level of consciousness (sometimes accompanied by convulsions), neurocognitive symptoms, emotional lability, irritability, and fatigue [24–26]
5	Brain	Epilepsy, stroke, and respiratory center damage [27]
6	Kidneys	Acute renal failure, embolism, and thrombosis [28]
7	Skin	Papular stain, vesicular rash, erythema (pseudo frostbite of hands and feet), “covid feet,” urticaria [11, 29], chickenpox, varicelliform vesicles [30], furunculosis, acne, eczema, dermatitis, and hair loss [15, 24–26]

**Table 2:** Potential medicinal plants for the treatment and prevention of COVID-19.

No.	Plant name	Biological activity
1	*Nigella sativa*	Anti-inflammation, analgesic [44], hypoglycemic, antihypertensive, antiasthmatic, antimicrobial, antiparasitic, antioxidant, anticancer [45], and immune-stimulating effects [46]
2	*Glycyrrhiza spp*. (*Glycyrrhiza glabra*, *Glycyrrhiza uralensis*)	Antioxidant, anti-inflammatory, antitussive, antidiabetic, antiviral, anticancer, antimutagenic, antiulcer, hepatoprotective [47], respiratory, immunological, analgesic, antipyretic effects [48], skin anti-aging, photoprotective, hair care, and anti-acne [49]
3	*Artemisia annua*	Antibacterial, anti-inflammatory [50], therapeutic properties [51], analgesic effects [52], antioxidant, antimicrobial, antifungal, antiulcer, larvicidal, antiasthmatic, anticancer, antihyperlipidemic, antiepileptic, hepatoprotective, antimalarial, antidiabetic, antiparasitic, antidepressant, gastroprotective, and antiviral activities [51]
4	*Artemisia vulgaris*	Anticancer [53], antimalarial, analgesic [52], and gastroprotective [51]
5	*Colchicum autumnale*	Anti-inflammatory, antiviral properties [54], and antioxidant [55]
6	*Inula helenium*	Expectorant and anti-inflammatory effects [56, 57]
7	*Aloe vera*	Anti-inflammatory, antibacterial, choleretic, anti-burn, and wound healing properties [58]
8	*Filipendula*	Immunomodulatory, bracing effect, diuretic [59, 60], diaphoretic activity [61–65], anti-inflammatory, antioxidant, and gastroprotective activity [66]
9	*Chamaenerion angustifolium* Seg.	Anti-inflammatory, blood-thinning, antipyretic, analgesic, astringent effect [67], immunomodulatory, antimicrobial, antioxidant, antiproliferative effects [68], antiphlogistic, antiseptic [36, 69], antibacterial, antifungal, and antiviral [70]

**Table 3:** Pharmacological activity of *Chamaenerion angustifolium* Seg.

No.	Symptoms COVID-19/post-covid symptoms	Chemical substance	Pharmacological activity
1	Pulmonary fibrosis [15]	Chlorophyll, phytosterols, vitamin C, tannins, carotenoids, organic acids, and copper	Wound healing action: accelerates the processes of epithelization and granulation of damaged tissues
2	Blood thickening, thrombosis, and embolisms [13]	Coumarins, vitamin C	Blood-thinning effect
3	Cytokine storm, body temperature rise, chills, fever, conjunctivitis [119]	Flavonoids	Anti-inflammatory, antipyretic effect
4	Impaired regulation of blood pressure (hypertensive crises, and hypotension) [119]	Magnesium, vitamin B, flavonoids	Normalizes blood pressure
5	Muscle and joint pain, chest pain and/or discomfort, and weakness [119]	Isoleucine	Analgesic effect
6	Headache [15, 119]	Thiamine, vitamin B2, vitamin B6, nicotinic acid	Analgesic effect
7	Gastrointestinal ulcers, inflammation of the mucosa of the stomach and intestines [119]	Tannin	Promotes healing of gastric and duodenal ulcers
8	Intoxication [24, 25]	Kaempferol, quercetin	Diuretic action is a powerful natural cleanser of various intoxications, including alcohol, chemical, and radiation pollution
9	Metabolic disorders [24, 25]	Chlorophyll	Improves metabolic processes in the body: increases the efficiency of absorption of nutrients and is involved in the regulation of carbohydrate-lipid metabolism. It affects diseases related to metabolic disorders
10	Fatigue [24, 26]	Polysaccharides	Restores strength in various kinds of exhaustion and fatigue
11	Disturbances of sleep, irritability [24, 26]	Magnesium, vitamin B, flavonoids, glycine	Recommended for overstimulation, migraine increased nervous overload, stress, neurosis, and insomnia. The tranquilizing and sedative effect relieves and eliminates depression
12	Epilepsy fit, confusion, decreased level of consciousness (sometimes accompanied by seizures), and neurocognitive symptoms [24, 26]	Arginine	Relieves symptoms of epilepsy, has an anticonvulsant effect
13	Nausea, vomiting, and diarrhea [24, 25]	Polysaccharides	It has a softening, enveloping, and astringent effect
14	Myocarditis, heart rhythm disorders, and acute heart failure [13, 119]	Proline	It is indicated in case of vegetovascular dystonia, is used in case of cardioneurosis, neurocirculatory dystonia
15	Various types of skin rashes and skin diseases: furunculosis, acne eruption, eczema, dermatitis, and hair loss [29, 119]	Chlorophyll, phytosterols, vitamin C, tannins, carotenoids, organic acids, copper	In folk medicine, it is sometimes used to treat diathesis, psoriasis, gout, and salt metabolism disorders. It has cosmetic properties and strengthens the hair
16	Sore throat, dry and wet cough [15, 120]	Vitamin B2, chlorophyll, phytosterols, vitamin C, tannins, carotenoids, organic acids, copper	Anti-inflammatory effect; expectorant action, used for sore throat, upper respiratory tract catarrh, tonsillitis, tuberculosis, bronchopulmonary pathology
17	Psychological disorders (depression, emotional lability, anxiety) [24, 26]	Arginine	Reduces anxiety-depressive disorders and tension. Not addictive
18	Hypoxemia [121]	Group B vitamins, iron	It has a pronounced hematopoietic effect, improves blood quality, and increases the level of saturation

**Table 4:** Comparison between the effectiveness of the plants and the symptomatic therapy of COVID-19.

Symptom	Medicinal plant								
	*Chamaenerion angustifolium* Seg.	*Glycyrrhiza spp.*	*Nigella sativa*	*Artemisia annua*	*Artemisia vulgaris*	*Inula helenium*	*Aloe vera*	*Colchicum autumnale*	*Filipendula ulmaria*
Pulmonary fibrosis [15]	V	—	—	—	—	—	—	—	—
Blood thickening, thrombosis, and embolisms [13]	V	—	—	—	—	—	—	—	V
Cytokine storm, body temperature rise, chills, fever, and conjunctivitis [119]	V	V	—	—	—	—	—	—	—
Impaired regulation of blood pressure (hypertensive crises, and hypotension) [119]	V	V	V	—	—	—	—	V	V
Muscle and joint pain, chest pain and/or discomfort, and weakness [119]	V	—	—	—	—	—	—	V	V
Headache [15, 119]	V	—	V	V	V	—	—	V	V
Gastrointestinal ulcers, inflammation of the mucosa of the stomach and intestines [119]	V	—	V	V	V	V	V	V	V
Intoxication [24, 25]	V	—	V	—	—	—	—	V	V
Metabolic disorders [24, 25]	V	—	—	—	—	—	—	—	—
Fatigue [24, 26].	V	—	V	V	V	—	—	—	V
Disturbances of sleep, irritability [24, 26]	V	—	V	V	V	—	—	—	V
Epilepsy fit, confusion, decreased level of consciousness (sometimes accompanied by seizures), and neurocognitive symptoms [24, 26]	V	—	V	—	V	—	—	—	V
Nausea, vomiting, and diarrhea [24, 25]	V	—	—	—	V	—	—	V	V
Myocarditis, heart rhythm disorders, acute and heart failure [13, 119]	V	V	—	—	—	—	—	—	—
Various types of skin rashes, skin diseases: furunculosis, acne eruption, eczema, dermatitis, and hair loss [29, 119]	V	—	V	V	V	—	V	—	V
Sore throat, dry, and wet cough [15, 120]	V	V	—	—	V	V	—	—	V
Psychological disorders (depression, emotional lability, and anxiety) [24, 26]	V	—	V	V	V	—	—	—	V
Hypoxemia [121]	V	—	—	—	—	—	—	V	V

“V”- marked therapeutic effect; “—”- no therapeutic effect was recorded.
